# Left ventricular outflow tract stenting in late presenting transposition physiology with ventricular shunt and pulmonary stenosis: a case series

**DOI:** 10.3389/fcvm.2026.1717159

**Published:** 2026-03-26

**Authors:** Radityo Prakoso, Yovi Kurniawati, Sisca Natalia Siagian, Aditya Agita Sembiring, Damba Dwisepto Aulia Sakti, Brian Mendel, Olfi Lelya, Oktavia Lilyasari

**Affiliations:** 1 Division of Pediatric Cardiology and Congenital Heart Disease, Department of Cardiology and Vascular Medicine, National Cardiovascular Center Harapan Kita, Universitas Indonesia, Jakarta, Indonesia; 2 Department of Cardiology and Vascular Medicine, National Cardiovascular Center Harapan Kita, Universitas Indonesia, Jakarta, Indonesia

**Keywords:** d-TGA, LVOT stenting, palliative intervention, pulmonary stenosis, ventricular septal defect

## Abstract

Left ventricular outflow tract (LVOT) stenting is a palliative strategy for late-presenting d-transposition of the great arteries (d-TGA) with ventricular septal defect (VSD) and severe LVOT obstruction (LVOTO), particularly in settings with limited surgical resources. We reviewed six patients who underwent LVOT stenting at our center. Median age at intervention was 6 months (Q1–Q3: 25 days–18 months) and median weight was 4.0 kg (Q1–Q3: 3.3–5.1 kg). Median stent length was 23 mm (Q1-Q3, 15–29 mm). Median pre-procedural arterial oxygen saturation was 53.5% (Q1–Q3: 51–65%), increasing to median 85.5% (Q1–Q3: 84–89%) following stent implantation. All procedures were technically successful, with no major intraprocedural complications. Median follow-up duration was 6 months (Q1–Q3: 4 days–6 months). Two patients died during follow-up (at 4 days and 6 months), four proceeded to bidirectional cavopulmonary shunt (BCPS) as part of a Fontan pathway, and one had undefined outcomes. No patient achieved biventricular repair. In this series, LVOT stenting provided effective short-term relief of obstruction and improved systemic oxygenation but functioned primarily as a bridge toward single-ventricle (Fontan) palliation, rather than toward biventricular repair.

## 1 Introduction

Transposition physiology, characterized by ventriculoarterial discordance, includes a spectrum of congenital heart defects such as transposition of the great arteries (TGA), sometimes associated with ventricular septal defect (VSD) and left ventricular outflow tract obstruction (LVOTO) (1, 2). The systemic circulation is sustained by the functional capacity of the systemic right ventricle. In cases of transposition of the great arteries with left ventricular outflow tract obstruction (LVOTO), pulmonary circulation relies on the left ventricle's ability to propel blood through the narrowed pulmonary arteries. As the degree of obstruction increases, pulmonary blood flow becomes progressively impaired, resulting in worsening heart failure, growth retardation, and multiorgan dysfunction (3, 4).

The situation becomes even more complex in late-presenting patients, who often arrive beyond the optimal window for early repair. These patients may present with longstanding cyanosis or significant end-organ dysfunction, all of which increase the risk of conventional surgical intervention (5–7). In such high-risk settings, palliative transcatheter LVOT stenting has emerged as a viable alternative to temporarily relieve pulmonary obstruction and stabilize the patient. This intervention may serve as a bridge to later surgical repair, allowing time for ventricular recovery, growth, and optimization of comorbid conditions (4). In this setting, stenting the LVOT can restore antegrade pulmonary blood flow and acutely improve left ventricular function; however, it may also increase pulmonary artery pressure, and therefore does not necessarily redirect the physiology toward a biventricular pathway. In many late-presenting patients, particularly in resource-limited environments, palliation rather than definitive biventricular repair is often the only realistic therapeutic strategy.

Although experience remains limited and technically demanding, early reports suggest that LVOT stenting is feasible and may offer significant short-term hemodynamic benefit (3, 4). However, data on procedural safety, patient selection, and long-term outcomes remain sparse, particularly in late presenters from resource-limited settings. This case series aims to present our experience with LVOT stenting in late-presenting patients with transposition physiology, focusing on procedural techniques, early outcomes, and the potential role of this strategy in the broader context of staged or delayed repair.

## 2 Methods

### 2.1 Study population

Six patients with uncorrected transposition physiology complicated by severe left ventricular outflow tract obstruction (LVOTO) and ventricular septal defect (VSD), leading to profound systemic desaturation, were performed LVOT stenting in the period between July 2021 and May 2024. In our center, the primary indications for LVOT stenting were: (1) profound hypoxemia (typically arterial saturation <40%–50%), (2) impaired left ventricular systolic function (ejection fraction <40%), and (3) elevated surgical risk (5). There was no LVOTO Vmax cutoff on echocardiography used in this study.

### 2.2 Procedural details

**Figure 1:**
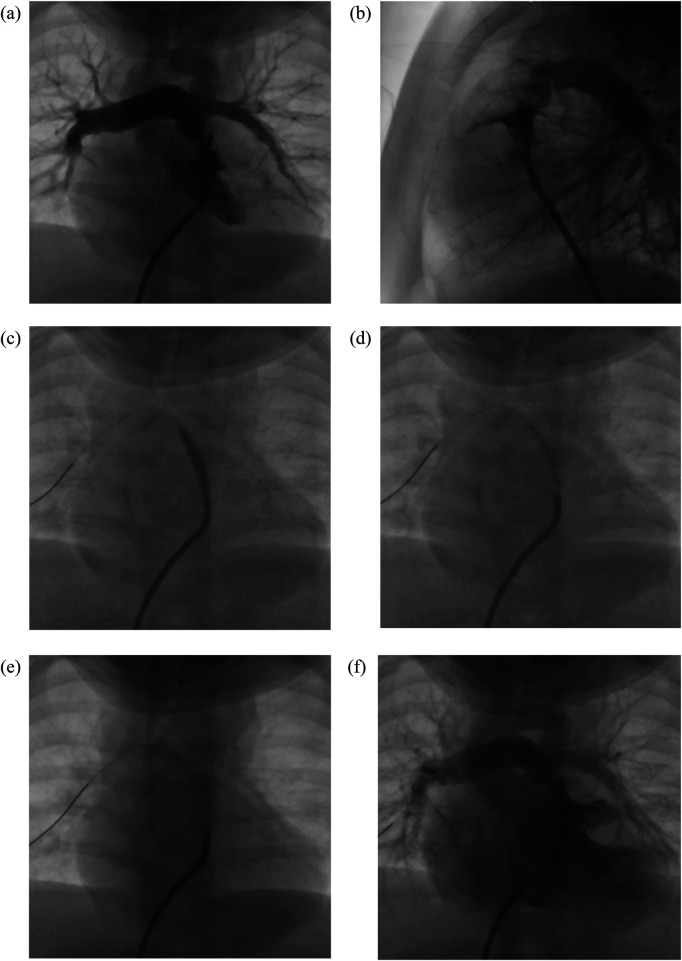
Left ventricular outflow tract (LVOT) stenting in a 6-month-old infant (body weight 5.1 kg) with d-transposition of the great arteries (d-TGA), complete atrioventricular septal defect (CAVSD), severe infundibular pulmonary stenosis, mesocardia, and right atrial isomerism. **(a, b)** Baseline angiogram demonstrating severe infundibular stenosis of the LVOT. **(c)** Balloon predilatation using an Emerge balloon 3.0 × 15 mm introduced via a JR guiding catheter 3.5/5F, supported by a Runthrough floppy wire positioned across the LVOT. The balloon was inflated at 14 atm for 10 s, repeated 20 times. **(d)** Deployment of a Resolute Integrity stent 4.0 × 15 mm through a JR guiding catheter 3.5/5F. **(e)** Fluoroscopic image showing the stent in position within the LVOT. **(f)** Post-stent LVOT angiography (cranial 27°, lateral view, 20 cc contrast) confirming stent patency with contrast opacification of the main pulmonary artery, left pulmonary artery (LPA), and right pulmonary artery (RPA), with good arborization and balanced pulmonary blood flow bilaterally.

All procedures were performed under sedation with mechanical ventilation. Patients were positioned supine with arms elevated to facilitate biplane angiography. Active warming devices were employed to prevent hypothermia, and a sterile field was established over the chest and upper abdomen. Vascular access was obtained via the right femoral vein or the right internal jugular vein. A Judkins Right (JR) guiding catheter was advanced into the LVOT over a 0.014” or 0.035” coronary wire. Left ventricular angiography was performed with the guiding catheter positioned at the outflow tract, utilizing a 30° left anterior oblique (LAO) and 30° cranial angulation, as well as a straight lateral projection (Figures 1a, b). Systemic anticoagulation was achieved with intravenous heparin (50 IU/kg body weight) (5–7).

Stent size and type were determined by Kirklin's full size, patient body weight, and LVOT stenosis length. Vascular stents were used in adolescents and adults, while coronary stents were employed in young children. Stent diameter was selected 1–2 mm larger than the diastolic infundibular measurement, with length sufficient to cover the distal muscular LVOT, pulmonary valve, and/or proximal main pulmonary artery before bifurcation (5–7).

Following stent selection, an appropriate delivery sheath or guiding catheter was advanced into the inferior vena cava. A 3.5–5F Judkins Right catheter was introduced through the sheath, and a rotating hemostatic valve was attached to its hub, connected to both the pressure line and contrast syringe. A 0.014”–0.035” coronary wire was advanced through the valve to the catheter tip. After system withdrawal, the left ventricle was re-entered under continuous pressure monitoring, and the LVOT was accessed with the catheter. Side-arm contrast injections confirmed the stenotic segment, and the coronary wire was advanced into a distal pulmonary artery branch (5–7).

The coronary wire was positioned across the lesion, and the pre-mounted stent was advanced to the intended LVOT landing zone. Side-arm contrast injections were repeated to confirm positioning. Predilatation with a coronary or vascular balloon was performed when required, particularly in cases of multilayer stenosis (Figure 1c). Stent deployment was achieved by manual balloon inflation, with simultaneous stabilization of the stent system (Figure 1d). The balloon was then gradually deflated while the delivery sheath was advanced over it to re-sheath securely within the stent (Figure 1e). Post-implantation management included intravenous furosemide (1 mg/kg) and a continuous milrinone infusion (0.375 μg/kg/min). Final assessment was performed with repeat LVOT angiography and transthoracic echocardiography (Figure 1f) (5–7).

### 2.3 Follow-up protocol

All patients were followed longitudinally from the time of LVOT stent implantation until their most recent documented clinical encounter. Follow-up data were obtained either through direct outpatient evaluations or by structured telephone interviews with caregivers when in-person visits were not feasible. During each follow-up, clinical status, oxygen saturation, occurrence of cyanotic spells, heart failure symptoms, and any stent-related complications (e.g., restenosis, migration) were assessed. Echocardiographic evaluation was performed at routine clinic visits to assess stent patency, ventricular function, and pulmonary artery flow. When patients did not return for scheduled evaluations, the most recent available clinical information, either from the last in-person consultation or telephone contact, was used to determine outcomes.

## 3 Results

LVOT stenting was performed in six patients with d-TGA, infundibular pulmonary stenosis, and ventricular shunt. Baseline characteristics are summarized in Table 1. All patients had elevated pre-procedural risk scores as assessed by the Catheterization RISk Score for Pediatrics (CRISP) (2). A percutaneous transfemoral antegrade approach was employed in all cases. Stents used included Omnilink Elite™ Stent (Abbott Vascular, Santa Clara, CA, USA) (*n* = 2), Promus PREMIER stent (Boston Scientific, Natick, MA, USA) (*n* = 1), Resolute Integrity™ Stent (Medtronic, Minneapolis, MN, USA) (*n* = 1), Resolute Onyx™ Stent (Medtronic, Minneapolis, MN, USA) (*n* = 1), and Dynamic™ Stent (Biotronik, Berlin, Germany) (*n* = 1). Stent placement was technically successful in all patients.

**Table 1:** Baseline characteristics of patient who had LVOT stenting.

No	Gender	Age	Diagnosis	Born weight (kg)	Body weight (kg)	Approach	Stent name	Stent diameter (mm)	Stent length (mm)	Saturation before (%)	Saturation during follow-up (%)	Follow-up duration (months)	Complication and recent follow-up
1	M	13 days	D-TGA, non-commited muscular inlet VSD, severe infundibular PS, ASD L-to-R shunt	2.9	2.8	Antegrade transfemoral	Promus PREMIER stent (Boston Scientific, Natick, MA, USA)	4	24	20	54	0	Death due to septic shock in ICU 4 day post procedure
2	M	25 days	D-TGA, restrictive VSD, severe infundibular PS, PFO, PDA L-to-R shunt, bilateral SVC	N/A	3.3	Antegrade transfemoral	Omnilink Elite™ Stent (Abbott Vascular, Santa Clara, CA, USA)	7	29	51	89	8	Death 6 months after bilateral BCPS
3	M	36 months	D-TGA, VSD, infundibular PS, dextrocardia, situs inversus	3	11.8	Antegrade transfemoral	Omnilink Elite™ Stent (Abbott Vascular, Santa Clara, CA, USA)	8	29	77	99	Lose to follow-up	None
4	F	6 months	D-TGA, CAVSD, severe infundibular PS, mesocardia, RA isomerism	3.3	5.1	Antegrade transfemoral	Resolute Integrity™ Stent (Medtronic, Minneapolis, MN, USA)	4	15	52	84	35	None, patient had undergone bilateral BCPS
5	M	18 months	D-TGA, subpulmonic VSD, severe valvar-subvalvar PS, bidirectional shunt PDA	3.9	4.3	Antegrade transfemoral	Resolute Onyx™ Stent (Medtronic, Minneapolis, MN, USA)	4.5	22	65	87	23	Patient had undergone bilateral BCPS, and evacuation of and LVOT stent.
6	M	2 months	D-TGA, muscular-inlet VSD, severe infundibular PS, RA isomerism, tricuspid atresia, right aortic arch, ASD R-to-L shunt	3	3.7	Antegrade transfemoral	Dynamic™ Stent (Biotronik, Berlin, Germany)	5	15	55	84	19	None, patient had undergone BCPS 1 month post LVOT stent

Patient 1 underwent successful implantation Promus PREMIER stent (Boston Scientific, Natick, MA, USA) 4.0 × 24 mm, with oxygen saturation improving from 20% to 54%. The procedure was uneventful; however, the patient died four days later. In the intensive care unit, the patient developed fever, leukopenia, and thrombocytopenia. Blood cultures grew Klebsiella pneumoniae. Despite supportive management and cardiopulmonary resuscitation following clinical deterioration and recurrent desaturation to 20%, the patient ultimately died. Patient 2 underwent successful LVOT stenting with an Omnilink Elite 7.0 × 29 mm stent but died following bilateral bidirectional cavopulmonary shunt (BCPS) surgery performed six months later.

## 4 Discussion

In transposition of the great arteries (TGA), the aorta arises from the right ventricle and the pulmonary artery from the left ventricle. In the presence of a ventricular septal defect (VSD) and left ventricular outflow tract obstruction (LVOTO), left ventricular outflow to the pulmonary artery is restricted, resulting in preferential shunting of blood across the VSD into the right ventricle and subsequently into the aorta. This physiology resembles that of tetralogy of Fallot rather than simple TGA (1, 5, 7). In neonates and young infants, several conventional strategies remain relevant. When atrial mixing is inadequate, balloon atrial septostomy can improve systemic oxygenation (8), while ductal stenting can provide a reliable source of pulmonary blood flow in cases where ductal patency is physiologically important (9). Alternatively, a systemic-to-pulmonary shunt offers another established means of augmenting pulmonary perfusion (10). Arterial switch may still be feasible in highly selected cases, although decision-making is complex in late presenters with significant LVOTO (11). Surgical options such as the Rastelli (12), REV (13), Nikaidoh (14), or Damus–Kaye–Stansel procedures (15) also represent potential pathways to repair; although a prior LVOT stent may theoretically complicate these operations, it does not necessarily preclude them. In our cohort, several patients ultimately proceeded to BCPS, reflecting a single-ventricle trajectory that, in resource-limited settings, may serve as a deliberate and appropriate destination strategy rather than a compromise. Pursuit of a two-ventricle repair is not always advisable, particularly when comorbidities, anatomy, or institutional resources introduce excessive risk. Thus, LVOT stenting should be viewed within the broader landscape of adapted, context-specific management, with individualized decisions balancing physiology, safety, and the realities of the healthcare environment.

Left ventricular outflow tract (LVOT) stenting in transposition patient with ventricular shunt and LVOTO is conceptually analogous to right ventricular outflow tract (RVOT) stenting, with the primary aim of re-establishing antegrade blood flow and optimizing pulmonary circulation. By securing flow across the obstructed LVOT, this strategy stabilizes pulmonary hemodynamics, improves ventricular performance, and ensures adequate forward cardiac output (5–7). Compared with RVOT stenting, however, LVOT stenting carries a higher risk of coronary artery compression, warranting pre-procedural coronary computed tomography evaluation. The technical challenges of LVOT stenting, however, are greater than those of RVOT stenting. In contrast to the right ventricular outflow tract (RVOT), which typically possesses a well-defined infundibulum, the left ventricular outflow tract (LVOT) generally lacks this anatomical feature. Consequently, stent placement is often performed across the pulmonary valve, thereby limiting subsequent surgical options and directing the patient toward a single-ventricle palliation strategy.

Liao et al. (2022) (3) reported successful LVOT stenting in an 8-year-old girl following repair of double-outlet right ventricle (DORV). An 8-zig, 2.2-cm CP stent (Numed Inc., Hopkinton, NY, USA) mounted on a 12-mm balloon-in-balloon catheter was deployed. Post-procedure, the patient's exercise tolerance improved, with functional status increasing from New York Heart Association (NYHA) class III to class II.

McMahon et al. (2013) (4) described LVOT stenting in a 7-week-old infant weighing 2.8 kg with mitral atresia, hypoplastic left ventricle, VSD, d-TGA, severe pulmonary stenosis, and hypoplastic branch pulmonary arteries. In this case, LVOT stenting was selected because either a modified Blalock–Taussig–Thomas (mBTT) shunt or a central shunt carried the risk of further distorting the already hypoplastic pulmonary arteries. The arterial duct was markedly tortuous and deemed unsuitable for stenting. Moreover, given the patient's low birth weight and the presence of hypoplastic pulmonary arteries with antegrade pulmonary blood flow, shunting was considered likely to result in pulmonary overcirculation.

Alternative devices have also been explored for use in infants and small children, including bioresorbable scaffolds. However, early experience with the Magmaris® Resorbable Magnesium Scaffold (RMS; BIOTRONIK AG, Switzerland) has raised important safety concerns. In one report, premature scaffold collapse occurred as early as two weeks after implantation, underscoring the need for vigilant post-procedural monitoring. This failure is thought to be related either to accelerated scaffold resorption in very young infants or to insufficient radial strength of RMS to counteract acute vessel recoil, ultimately resulting in inadequate relief of branch pulmonary artery stenosis (16).

More recently, newer stent systems specifically engineered for pediatric use have shown promising results. The Minima Stent System represents the first device designed, tested, and approved by the FDA for neonates, infants, and children. In a cohort of 42 patients, including those with branch pulmonary artery stenosis (PAS), recurrent coarctation (CoA), and native CoA, Minima implantation achieved a 97.6% success rate. Patients had a median age of 9 months and weight of 7.8 kg, with stenting yielding a median 131% increase in minimal vessel diameter and a significant reduction in pressure gradients in CoA patients (from 25 mmHg to 0 mmHg) (17).

Similarly, the Optimus-L stent has demonstrated feasibility in small children requiring a low-profile approach. In a series of 28 patients (median age 3.4 years, median weight 12.9 kg), including infants and children ≤10 kg, Optimus-L stents were manually crimped onto small balloon catheters (≤12 mm) and delivered through ≤8 Fr sheaths. The stents were successfully implanted across a variety of stenotic lesions, including branch pulmonary arteries, the aortic isthmus, right ventricular outflow tract, and Glenn anastomosis, without procedural complications. These findings support the Optimus-L as a safe and effective option for treating both arterial and venous stenoses in younger, smaller patients (18).

Post-stenting repair after LVOT stenting remains limited, as the stent is often positioned across the pulmonary valve and subpulmonary region, potentially distorting valve anatomy, restricting leaflet motion, and complicating subsequent intracardiac rerouting; the scaffold may also embed into surrounding tissue, increasing operative difficulty. As a result, LVOT stenting in late-presenting transposition physiology functions primarily as a palliative or bridging measure rather than a pathway to biventricular repair, with definitive reconstruction feasible only in highly selected cases. Management of pulmonary artery flow after stenting is equally critical: while restored antegrade flow improves oxygenation, it also risks pulmonary overcirculation. To maintain balanced perfusion, we employed close hemodynamic and echocardiographic monitoring, early diuretic therapy, and milrinone support, with adjustments in ventilator settings, fluids, and inotropic therapy as needed to prevent both overflow and inadequate pulmonary blood flow during the early post-procedural period.

## 5 Limitations

A potential drawback of LVOT stenting is its impact on subsequent surgical repair. The rigid stent may distort the geometry of the subpulmonary region or the pulmonary valve, thereby complicating later intracardiac rerouting procedures. In addition, stent struts may embed within the endocardium or fibrose into surrounding tissue, requiring surgical excision or necessitating modifications. These factors can increase operative complexity and risk.

## 6 Conclusions

Our case series highlights LVOT stenting as an alternative to ductal stenting or surgical systemic-to-pulmonary shunt in children with this anatomy. This minimally invasive approach stabilizes antegrade pulsatile pulmonary blood flow and promotes pulmonary arterial perfusion and growth, thereby facilitating subsequent procedures.

## Data Availability

The original contributions presented in the study are included in the article/Supplementary Material, further inquiries can be directed to the corresponding author.
